# Migration of surgical clips through a right lobectomy stump mimicking an asthmatic syndrome

**DOI:** 10.1186/1471-2482-13-S2-S32

**Published:** 2013-10-08

**Authors:** Vincenzo Di Crescenzo, Paolo Laperuta, Filomena Napolitano, Chiara Carlomagno, Michele Danzi, Bruno Amato, Alfredo Garzi, Mario Vitale

**Affiliations:** 1 Department of Medicine and Surgery, University of Salerno, Italy; 2 Department of Clinical Medicine and Surgery, University of Naples "Federico II", Italy

**Keywords:** surgical clips, bronchial stump, fistula

## Abstract

The mechanical stapler is routinely used in thoracic surgery practice to attend resection of bronchus and vessels. Herein, we reported a very rare complication as the migration of a titanium surgical clip through a right lobectomy stump. One year after the procedure, the patient complained of persistent cough. A misdiagnosis of asthma was made and she treated for 6 months with bronchodilators, corticosteroid and antihistaminic without success. Thus, patient re-referred of our unit. No clinical signs of infection as fewer, productive cough, dyspnea were present. The laboratory exams were within normal value including white cells. CT scan revealed no abnormalities. Bronchoscopy demonstrated a healed upper bronchus stump without evidence of an actual, open bronchopleural fistula but with clips apparently working their way into the airway, with approximately half of the clip visible within the lumen. The side of the clips that would be open before closure by the surgeon formed the leading edge of the clips visible in the lumen. The clips were successfully removed during flexible bronchoscopy with a forceps usually used for biopsy. After the procedure, the cough disappeared. The endoscopy check after 3 months showed a normal bronchial stump without evidence of fistula.

## Background

The mechanical stapler is routinely used in thoracic surgery practice to attend resection of bronchus and vessels. Herein, we reported a very rare complication as the migration of a titanium surgical clip through a right lobectomy stump. One year after the procedure, the patient complained of persistent cough. A misdiagnosis of asthma was made and she treated for 6 months with bronchodilators, corticosteroid and antihistaminic. Symptoms disappeared after removing surgical clip with flexible bronchoscopy.

## Clinical presentation

**Figure 1:**
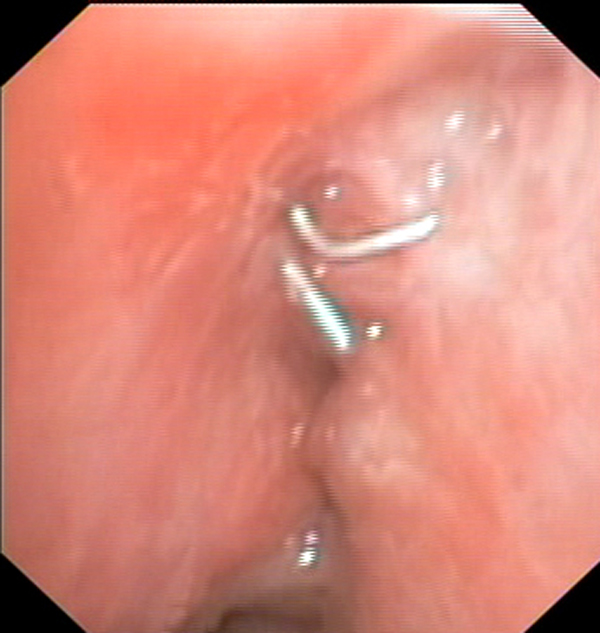
**The endoscopic view showed the migration of clips from the bronchial stump into the airway**.

A 75 year-old woman patient was referred to our unit for the diagnosis and treatment of lung lesion discovered at Chest/X ray. She complained of cough and fewer. All laboratory exams were within normal values. Chest tomography scan diagnosed the presence of lesion (size 3 cm) confined within upper right lobe without lymp node involvement. On PET scan, the lesion was FDG- avid with a standard uptake value (SUV) of 3.5. No other lesions were found. Fine-needle aspiration biopsy diagnosed (FNAB) CT guided diagnosed to be a atypical carcinoid. Cardiorespiratory evaluation did not contraindicate surgery. Thus, a right upper lobectomy via thoracotomy was attended in a standard matter. The bronchus was mechanically closed using a mechanical stapler. A radical lymph adenectomy was performed. No complications occurred during operation and in the postoperative course. In addition to peridural analgesia, a transcutaneous electrical nerve stimulation analgesia was applied to better control the postoperative pain [1]. The patient was discharged 7 days after operation. The pathological diagnosis confirmed the tumor to be an atypical carcinoid without pathological lymph node.(pathological stage T2N0M0). Follow-up revealed no recurrence. However, after 1 year from the procedure the patient complained of persistent cough. Supposing a diagnosis of asthma, a therapy with steroid and histaminic was started and continued for 6 months without success. Thus, patient re-referred of our unit. No clinical signs of infection as fewer, productive cough, dyspnea were present. The laboratory exams were within normal value including white cells. CT scan revealed no abnormalities. Bronchoscopy demonstrated a healed upper bronchus stump without evidence of an actual, open bronchopleural fistula but with clips apparently working their way into the airway, with approximately half of the clip visible within the lumen. The side of the clips that would be open before closure by the surgeon formed the leading edge of the clips visible in the lumen (Figure 1). The clips were successfully removed during flexible bronchoscopy with a forceps usually used for biopsy. After the procedure, the cough disappeared. The endoscopy check after 3 months showed a normal bronchial stump without evidence of fistula.

## Discussion

Foreign body erosion into the tracheobronchial tree is uncommon and its presentation is highly variable. In literature few reports reported the expectoration of staples and dry bovine pericardial strips used for staple line reinforcement after surgery for emphysema [2,3]. Saunders and coworkers [4] reported a case of bullet migration through pulmonary parenchyma and its spontaneous expulsion. Only one paper from Ahmed et al [5] reported a migration of clips from pneumonectomy and spontaneously expectorated.

In the present case the persistent cough, appeared after 1 year from the lobectomy, was misdiagnosed as asthma disease. The patient was treated for 6 months with medical therapy including bronchodilators, corticosteroid and antihistaminic without success. Then, she was re-referred to our unit. Despite the presence of cough, the diagnosis of bronchopleural fistula was unlike due to lack of fewer, of productive cough and of other clinical and laboratory signs of infection. Bronchoscopy showed a healed upper bronchus stump without evidence of an actual, open bronchopleural fistula but with clips apparently working their way into the airway, with approximately half of the clip visible within the lumen. The side of the clips that would be open before closure by the surgeon formed the leading edge of the clips visible in the lumen. Thus, the persistent cough was due to chronic irritation of bronchial mucosa by clips. Conversely to Ahmed et al [5] who decided against to remove the clips, we decided to take away the clips because the patient was symptomatic. Despite rigid bronchoscopy is usually indicated for removing foreign body obstructing air way [6-16], in the present case the clips were successfully extracted with a flexible bronchoscopy. After the procedure, the persistent cough disappeared. Yet, the endoscopic view at 3 months showed a normal bronchial stump. In conclusion, in patient undergoing lung resection with persistent cough, bronchoscopy is mandatory in order to exclude the presence of bronchopleural fistula and/ or the migration of surgical clips used to attend bronchus or vessels ligation.

## Competing interests

The authors declare that they have no competing interests.

## Authors' contributions

P.L. : conception and design, interpetration of data, given final approval of the version to be published. F. N.: acquisition of data, drafting the manuscript, given final approval of the version to be published. C.C. : acquisition of data, drafting the manuscript, given final approval of the version to be published. MD : acquisition of data, drafting the manuscript, given final approval of the version to be published. BA : acquisition of data, drafting the manuscript, given final approval of the version to be published. A.G. : acquisition of data, drafting the manuscript, given final approval of the version to be published. M.V. : acquisition of data, drafting the manuscript, given final approval of the version to be published. V.D.C.: critical revision, interpretation of data, given final approval of the version to be published.

## Authors' information

PL: Resident at of Thoracic Surgery - University of Salerno. FN: Resident at of Thoracic Surgery - University of Salerno. CC: Resident in Department of Clinical Medicine and Surgery -University of Naples. MD : acquisition of data, drafting the manuscript, given final approval of the version to be published. BA : acquisition of data, drafting the manuscript, given final approval of the version to be published. AG: Assistant Professor of Pediatric Surgery - University of Salerno. MV: Associate Professor of Endocrinology - University of Salerno. VDC: Assistant Professor of Thoracic Surgery - University of Salerno.

## Declarations

Funding for publication of this article was funded by a donation given by Merck Serono spa to the Department of Clinical Medicine and Surgery, University of Naples "Federico II", Italy.

This article has been published as part of *BMC Surgery *Volume 13 Supplement 2, 2013: Proceedings from the 26th National Congress of the Italian Society of Geriatric Surgery. The full contents of the supplement are available online at http://www.biomedcentral.com/bmcsurg/supplements/13/S2
